# Immunosenescence markers in T- and NK-cells according to the CD4/CD8 ratio in successfully treated people living with HIV

**DOI:** 10.3389/fmed.2025.1562537

**Published:** 2025-04-15

**Authors:** Matteo Vassallo, Jacques Durant, Sami Addou, Michel Ticchioni, Roxane Fabre, David Chirio, Alissa Naqvi, Eric Cua, Leslie Ameil, Maeva Godemert, Christian Pradier, Michel Carles

**Affiliations:** ^1^Internal Medicine/Infectious Diseases Department, Cannes, France; ^2^Infectious Diseases Department, University of Nice, Nice, France; ^3^Department of Immunology, University of Nice, Nice, France; ^4^Department of Public Health, University of Nice, Nice, France; ^5^Pain Department and FHU InovPain, CHU Nice and Cote Azur University, Nice, France; ^6^Clinical Research Department, University of Nice, Nice, France

**Keywords:** HIV, CD4/CD8 ratio, immunosenescence, comorbidity, immune activation

## Abstract

**Introduction:**

The CD4/CD8 ratio has emerged as a useful indicator of immune dysfunction and comorbid conditions in people living with HIV (PLWH). However, its optimal cut-off value is unclear. We explored the correlation between the CD4/CD8 ratio, immunosenescence markers and comorbid conditions.

**Methods:**

We prospectively included PLWH on successful and stable ART (antiretroviral therapy) > 60 years old and receiving either BIC/FTC/TAF or DTG/3TC, in Nice, France. HIV-negative healthy subjects were included as controls. We measured T-cell subsets (naïve, central memory, effector memory and terminally differentiated cells) and the distribution of KLRG1 + CD57+ senescent cells. We correlated CD4/CD8 ratio, background measurements and comorbid conditions.

**Results:**

We included 68 PLWH (median age 69 years, 31 years on ART, median CD4/CD8 ratio 0.76). PLWH had higher levels of senescence markers than controls (*n* = 8). Among PLWH, adjusting for age, gender, HIV follow-up and duration on ART, those with a CD4/CD8 ratio < 0.76 had more senescent CD8+ cells (AdjOR = 0.93, 95%CI = [0.88; 0.97], *p*-value = 0.003). Higher levels of CD8+ senescence persisted for lower CD4/CD8 ratios, with, in addition, a significant decrease in NK cells in case of a ratio < 0.4. After adjustment, CD8+ effector memory senescent cells were significantly more abundant in PLWH with hypertension.

**Conclusion:**

PLWH on successful ART display elevated immunosenescence markers, mainly on CD8+ T-cells. A CD4/CD8 cut-off value below 0.4 showed the strongest association with immune dysfunction, including NK+ cells. Such results could be useful for identifying patients requiring closer follow-up and screening for complications.

## Introduction

The CD4/CD8 ratio has emerged as a useful marker of immune dysfunction in people living with HIV (PLWH). Indeed, despite successful antiretroviral therapy (ART), PLWH continue experiencing more frequent non-AIDS clinical events and a higher mortality rate than uninfected subjects (1). The main current hypothesis is that HIV infection is associated with chronic inflammation, leading to immune activation and premature aging of the immune system, often referred to as immunosenescence (2, 3). Possible explanations for such chronic immune activation, despite successful control of HIV replication in plasma, include persistent low-level HIV replication, microbial translocation and co-infection (4).

A low CD4/CD8 ratio has been linked to immunosenescence and inflammation (5, 6). Indeed, the constant stimulation of the immune system by HIV, associated with thymus atrophy with age, could be responsible for T-cell exhaustion and a reduced CD4/CD8 ratio, mainly driven by the loss of CD4+ and the expansion of CD8+ cells (7). Moreover, the chronic immune activation during HIV is also responsible for NK cells dysfunction, which play a critical role in innate immunity and have been associated with disease progression (8).

The CD4/CD8 ratio has thus been associated with a spectrum of comorbidities, including cerebrovascular conditions, neurocognitive disorders, chronic kidney and obstructive pulmonary diseases and non-AIDS malignancies (9). However, the most informative cut-off values for predicting clinical events remain unclear. Indeed, some studies showed that values below 0.5 are associated with higher mortality (9), while others suggested that a cut-off value of 0.3 or a CD8 cell count >1,500, regardless of CD4 count, are predictors of clinical progression (1, 10).

The aim of this study was to explore immunosenenescence markers according to different CD4/CD8 ratio cut-off values among successfully treated PLWH aged over 60 years, in order to identify the best predictive value for complications.

## Methods

### Study design and participants

“Collateral” is a multicentre, on-going, French prospective cohort study, conducted in Nice and Cannes hospitals, in France, which includes participants over 40 years of age or on ART for over 10 years, and currently on stable and successful ART with either Bictegravir/Emtricitabine/Tenofovir Alafenamide (BIC/FTC/TAF) or Dolutegravir/Lamivudine (DTG/3TC) for at least 6 months. The main goal of the study is to explore inflammatory markers and comorbid conditions.

We performed a sub-study on PLWH over 60 years, aiming to measure immunosenescence markers and to correlate these with the CD4/CD8 ratio and comorbid conditions. Healthy subjects matched for age were also recruited.

Exclusion criteria were subjects with either chronic hepatitis B or C and those having archived mutations on DTG, BIC or TAF.

### Cellular markers of immune activation and senescence

Flow cytometric analysis was performed on blood collected on EDTA and labeled within 24 h. Briefly, immunophenotypical analysis was performed using 12-color flow cytometry (BD FACSLyric™, BD Biosciences, San Jose, CA). The following antibodies were used in this study: fluorescein isothiocyanate (FITC)-conjugated-CD57; phycoerythrin (PE)-conjugated-KLRG1; Peridinin chlorophyll protein-Cy5.5 (PerCP-Cy5.5)-conjugated-CD8; PE-Cy7-conjugated-CD27; allophycocyanin (APC)-conjugated-CD45RA; APC-H7-conjugated-HLA-DR; Brilliant Violet (BV)-conjugated-CD56; BV510-conjugated–CD3; BV605-conjugated–CD4, BV711-conjugated–CD38; BV786-conjugated-CD45, all purchased from BD Biosciences.

Instrument set up was performed according to the recommendations of the manufactor and France Flow Standard Operating Procedures (11).

Identification of lymphocytes was done using a combination of SSC/FSC properties and CD45 expression. The gating strategy for senescent cells was next based on the gating of CD3 + CD4+ and CD3 + CD8+ lymphocytes, and identification of CD28 negative, CD57 and KLRG1 positive cells. Sub-populations of naïve, memory, effector and effector memory T cells were identified according to the expression of CD45RA and CD27 (12–14).

Moreover, according to previous reports, cells expressing both the co-inhibitory Killer-cell Lectin-like Receptor 1 (KLRG-1) and CD57 markers were considered as senescent (15, 16). The prevalence of senescent cells among T-cell subsets was measured, in order to confirm their wider distribution among highly differentiated cells.

NK cells were defined as CD56 positive and CD3 and CD19 negative lymphocytes. An example of our strategy is described in Supplementary Figure S1. We also explored NK KLRG-1 CD56^bright^ cells, an important NK cell subset known for its immunoregulatory properties (17).

Supplementary Figures S1, S2 show the gating strategy.

### Statistical analysis

The main background characteristics of patients were collected, i.e., age, years since HIV diagnosis, ART regimen, years on ART, CD4/CD8 ratio, nadir CD4 and type of comorbid conditions.

For each patient, frequencies and percentages were calculated for qualitative variables, while means and standard deviations were measured for quantitative parameters. The median CD4/CD8 ratio for the whole population was calculated and differences in immunosenescence markers were measured according to values below and above the median. The Shapiro test was used as quantitative test for normality.

Following these measurements, non-parametric tests were performed. The Wilcoxon-Mann–Whitney-test was used to compare different CD4/CD8 cut-off values with immunosenescence markers in cell subsets. Either Khi2 or Fisher test were used for quantitative parameters. In case of factors with univariate *p* values < 0.05, multivariate models were then fitted.

Spearman’s rho correlation allowed comparisons between CD4/CD8 values as continuous variables and markers of immune activation and immunosenescence for CD4+, CD8+, and NK cells. Kruskal-Wallis tests were used in order to compare three groups: PWH with CD4/CD8 ratio below and above median values, and controls. In case of *p*-values ≤ 0.05, *post-hoc* Steel Dwaas tests were added. Analyses were performed using R-4.3.0 software.

## Results

### Study population

We included 68 PLWH (median age 69 years, 81% men, 31 years since known HIV infection, 25 years on ART, median CD4/CD8 ratio 0.76). Current ART consisted either in DTG/3TC (56% of PLWH) or in BIC/FTC/TAF (44%), with 2 years’ median treatment duration. Main comorbid conditions were high blood pressure (32%), osteoporosis (21%) and previous malignancy (18%) (Table 1).

**Table 1:** Patients characteristics at baseline.

	*N* (%) or median [Q1; Q3]
Number of patients	68
Age (years)	67.5 [63.4; 72.5]
Male gender	54 (81)
HIV follow-up (years)	30.7 [26.4; 34.0]
Time on ART (years)	25.3 [16.4; 26.9]
Time on DTG/3TC or TAF/FTC/BIC (years)	2.0 [1.4; 2.6]
CD4/CD8 ratio	0.76 [0.52; 1.07]
Comorbidities	
High blood pressure	22 (32)
Osteoporosis	14 (21)
Diabetes	9 (13)
Cancer	12 (18)

ART, antiretroviral therapy; DTG, Dolutegravir; 3TC, Lamivudine; BIC, Bictegravir; FTC, Emtricitabine; TAF, Tenofovir alafenamide.

Eight healthy controls, matched for age, were also included for comparison. Their CD4/CD8 ratio was above 1 for all subjects and significantly higher than among PLWH (data not shown).

### Markers of immunosenescence and T-cell subsets in PLWH and controls according to the CD4/CD8 ratio

As expected, the prevalence of senescent cells was higher among EM and TEMRA T-cell subsets (data not shown) and PLWH had higher levels of senescent markers than controls (Table 2).

**Table 2:** T-cell subsets and immunosenescence markers in controls and PLWH, according to their CD4/CD8 ratio median values.

	CD4/CD8									
	1—Controls		2—PLWH ≤ 0.76		3—PLWH > 0.76			*Post-hoc* tests		
	*n* = 8		*n* = 35		*n* = 33					
	Mean	[SD]	Mean	[SD]	Mean	[SD]	*p*-value^*^	1 vs. 2^**^	1 vs. 3^**^	2 vs. 3^**^
lymphocyte count (cells/microL)	1950.0	[325.1]	2005.1	[604.1]	2160.2	[701.7]	0.384			
CD3+ (% of lymphocytes) (%)	65.6	[14.0]	71.8	[8.6]	65.8	[11.0]	0.036	0.022	0,622	1,000
NK (%)	14.7	[6.3]	15.0	[8.2]	18.2	[10.0]	0.359			
CD4+ (%)	41.6	[11.2]	22.6	[5.7]	33.9	[8.9]	<0.001	<0.001	<0.001	<0.001
CD8+ (%)	20.6	[8.5]	45.9	[9.1]	27.7	[6.4]	<0.001	<0.001	<0.001	<0.001
CD8+ senescent (%)	24.4	[21.2]	47.9	[14.7]	31.8	[12.0]	<0.001	<0.001	<0.001	<0.001
CD4+ senescent (%)	1.9	[2.7]	9.9	[8.9]	6.7	[11.6]	0.003	0.086	0.007	0.074
NK senescent (%)	61.5	[14.5]	64.3	[18.2]	63.0	[20.7]	0.686			
NK senescent CD56^bright^ (%)	1.7	[1.8]	1.0	[1.0]	0.7	[0.7]	0.219			

PLWH, People living with HIV; Senescent cells, defined by the double surface expression of KLRG1 and CD57; ^*^Comparison between PLWH and controls with Kruskal-Wallis test. ^**^Steel Dwass *post-hoc* test.

**Figure 1:**
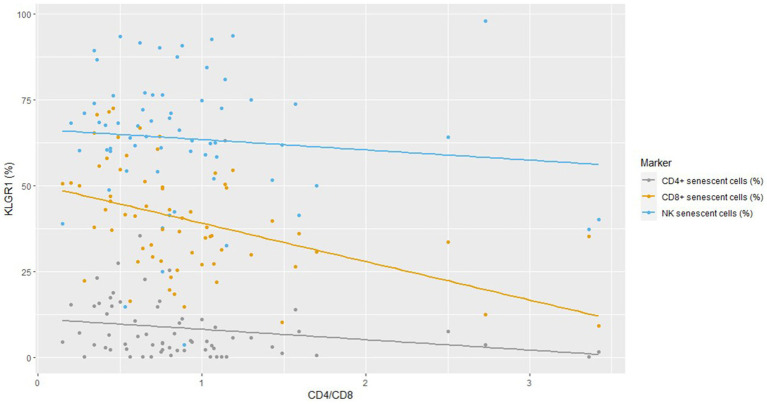
Correlation between KLRG1 expression on cell subsets and the CD4/CD8 ratio.

The strongest negative correlation between KLRG1 expression and the CD4/CD8 ratio was found for CD8+ cells (Figure 1) and Spearman’s rho correlation confirmed that the CD4/CD8 ratio was negatively associated with CD4+ and CD8+ senescent cells (Supplementary Table 1). No difference in immunosenescence was found between treatment with BIC/FTC/TAF and with DTG/3TC (data not shown).

According to median CD4/CD8 ratios, PLWH with values below 0.76 had more CD8+, senescent CD8+ cells, fewer CD4+ cells, and were more frequently men. After adjusting for age, gender, HIV follow-up and duration on ART, senescent CD8+ cells remained more abundant among those with a CD4/CD8 ratio below 0.76 (AdjOR = 0.93, 95%CI = [0.88; 0.97], *p*-value = 0.003), together with the number of CD8+ cells and, with reverse findings for the CD4+ cell count (Table 3).

**Table 3:** Comparisons between PWH according to different CD4/CD8 ratio cut-off values.

Cut-off value 0.76	CD4/CD8					
	≤0.76		>0.76			Adjusted
	*n* = 35		*n* = 33			
	Mean	[SD]	Mean	[SD]	*p*-value^*^	*p*-value^**^
Time on last ART (years)	2.2	[1.1]	2.1	[1.2]	0.846	
HIV follow-up (years)	27.2	[8.6]	29.6	[7.3]	0.308	
Age at inclusion	69.9	[6.3]	66.0	[6.5]	0.006	
Time on ART (years)	21.9	[7.9]	21.9	[6.7]	0.631	
lymphocytes count (cells/microL)	2005.1	[604.1]	2160.2	[701.7]	0.202	
CD3+ cells (%)	71.8	[8.6]	65.8	[11.0]	0.007	0.040
NK cells (%)	15.0	[8.2]	18.2	[10.0]	0.168	
CD4+ cells (%)	22,6	[5.7]	33.9	[8.9]	<0.001	0.000
CD8+ cells (%)	45.9	[9.1]	27.7	[6.4]	<0.001	0.003
senescent CD8+ cells (%)	47.9	[14.7]	31.8	[12.0]	<0.001	0.003
senescent CD4+ cells (%)	9,9	[8.9]	6.7	[11.6]	0.035	0.727
senescent NK cells (%)	64.3	[18.2]	63.0	[20.7]	0.755	
NK CD56^bright^ cells (%)	1.0	[1.0]	0.7	[0.7]	0.128	
	*n*	(%)	*n*	(%)	*p*-value^***^	
Gender					0.026	
Women	3	(23.1)	10	(76.9)		
Men	31	(57.4)	23	(42.6)		
Treatment with statins					1.000	
No	19	(50.0)	19	(50.0)		
Yes	13	(50.0)	13	(50.0)		

Cut-off value 0.6	CD4/CD8					
	≤0.6		>0.6			Adjusted
	*n* = 22		*n* = 46			
	Mean	[SD]	Mean	[SD]	*p*-value^*^	*p*-value^**^
Time on last ART (years)	2.1	[1.1]	2.2	[1.1]	0.939	
HIV follow-up (years)	26.3	[8.8]	29.3	[7.5]	0.180	
Age at inclusion	70.2	[5.4]	67.0	[7.0]	0.014	
Time on ART (years)	21.4	[8.8]	22.1	[6.6]	0.615	
lymphocytes count (cells/microL)	2090.0	[687.4]	2077.6	[645.8]	0.798	
CD3+ cells (%)	73.0	[7.4]	67.0	[10.9]	0.024	0.122
NK cells (%)	14.0	[6.9]	17.8	[9.9]	0.236	
CD4+ cells (%)	19.6	[4.3]	32.0	[8.3]	<0.001	0.002
CD8+ cells (%)	50.2	[8.4]	31.0	[8.0]	<0.001	0.004
senescent CD8+ cells (%)	50.2	[15.0]	35.3	[13.7]	<0.001	0.012
senescent CD4+ cells (%)	10.5	[7.9]	7.4	[11.2]	0.041	0.971
senescent NK cells (%)	63.1	[18.1]	64.0	[20.1]	0.692	
NK CD56^bright^ cells (%)	0.9	[0.8]	0.9	[0.9]	0.664	
	*n*	(%)	*n*	(%)	*p*-value^***^	
Gender					0.049	
Women	1	(7.7)	12	(92.3)		
Men	20	(37.0)	34	(63.0)		
Treatment with statins					0.631	
No	11	(28.9)	27	(71.1)		
Yes	9	(34.6)	17	(65.4)		

Cut-off value 0.5	CD4/CD8					
	≤0.5		>0.5			Adjusted
	*n* = 17		*n* = 51			
	Mean	[SD]	Mean	[SD]	*p*-value^*^	*p*-value^**^
Time on last ART (years)	2.1	[1.2]	2.2	[1.1]	0.723	
HIV follow-up (years)	25.6	[8.9]	29.4	[7.5]	0.090	
Age at inclusion	70.5	[5.6]	67.2	[6.8]	0.025	
Time on ART (years)	21.1	[8.8]	22.2	[6.8]	0.724	
lymphocytes count (cells/microL)	2150.0	[726.6]	2059.0	[634.3]	0.861	
CD3+ cells (%)	73.7	[7.7]	67.2	[10.6]	0.016	0.094
NK cells (%)	13.8	[7.5]	17.6	[9.6]	0.197	
CD4+ cells (%)	18.7	[3.9]	31.4	[8.3]	<0.001	0.004
CD8+ cells (%)	51.9	[8.3]	32.0	[8.4]	<0.001	0.013
senescent CD8+ cells (%)	52.7	[13.7]	35.7	[13.8]	<0.001	0.007
senescent CD4+ cells (%)	11.9	[7.9]	7.1	[10.8]	0.007	0.646
senescent NK cells (%)	66.5	[15.7]	62.7	[20.5]	0.716	
NK CD56^bright^ cells (%)	1.0	[0.8]	0.8	[0.9]	0.375	
	*n*	(%)	*n*	(%)	*p*-value^***^	
Gender					0.028	
Women	0	(0.0)	13	(100.0)		
Men	17	(31.5)	37	(68.5)		
Treatment with statins					0.957	
No	10	(26.3)	28	(73.7)		
Yes	7	(26.9)	19	(73.1)		

Cut-off value 0.4	CD4/CD8					
	≤0.4		>0.4			Adjusted
	*n* = 8		*n* = 60			
	Mean	[SD]	Mean	[SD]	*p*-value^*^	*p*-value^**^
Time on last ART (years)	2.0	[1.9]	2.2	[1.0]	0.561	
HIV follow-up (years)	28.9	[8.1]	28.3	[8.0]	0.969	
Age at inclusion	68.3	[4.2]	68.0	[6.9]	0.555	
Time on ART (years)	21.5	[10.3]	22.0	[6.9]	0.468	
lymphocytes count (cells/microL)	2075.0	[740.2]	2082.3	[647.7]	0.803	
CD3+ cells (%)	75.1	[7.3]	68.0	[10.4]	0.053	
NK cells (%)	10.4	[5.9]	17.4	[9.3]	0.031	0.054
CD4+ cells (%)	15.7	[3.3]	29.8	[8.5]	<0.001	0.063
CD8+ cells (%)	56.7	[8.3]	34.4	[9.8]	<0.001	0.009
senescent CD8+ cells (%)	50.4	[15.2]	38.6	[15.3]	0.040	0.089
senescent CD4+ cells (%)	10.5	[7.9]	8.1	[10.6]	0.195	
senescent NK cells (%)	69.6	[15.7]	62.9	[19.7]	0.351	
NK CD56^bright^ cells (%)	1.0	[1.1]	0.8	[0.8]	0.898	
	*n*	(%)	*n*	(%)	*p*-value^***^	
Gender					0.338	
Women	0	(0.0)	13	(100.0)		
Men	8	(14.8)	46	(85.2)		
Treatment with statins					0.128	
No	7	(18.4)	31	(81.6)		
Yes	1	(3.8)	25	(96.2)		

^*^Wilcoxon-Mann–Whitney test. ^**^Logistic regression adjusted for age, gender, HIV follow-up, and time on ART. ^***^Chi-2 test or Fisher exact test.

CD8+ senescent cells remained more abundant after adjustment, even in case of CD4/CD8 ratios below 0.6 (AdjOR = 0.94, 95%CI = [0.89; 0.98], *p*-value = 0.012), 0.5 (AdjOR = 0.92, 95%CI = [0.87; 0.98], *p*-value = 0.007) and 0.4 (AdjOR = 0.95, 95%CI = [0.90; 1.00], *p*-value = 0.089), while, in the latter case, there were significantly fewer NK+ cells (Table 3).

### Correlation between markers of immunosenescence and comorbid conditions

PLWH with hypertension had more CD8+ EM senescent cells, while those with osteoporosis had more naïve CD4+, naïve CD8+ and NK CD56^bright^ senescent cells. Multivariate analysis showed that CD8+ EM senescent cells remained significantly more abundant in those with hypertension (AdjOR = 1.23, 95%CI = [1.08; 1.45], *p*-value = 0.005) (Supplementary Table 2).

Neither cancer nor diabetes were associated with significant immunosenescence markers.

## Discussion

In a cohort of PLWH, we found major alterations of immunosenescence markers, despite stable and successful treatment. There were more changes within T-cell subsets in those with a CD4/CD8 ratio below 0.76, while a cut-off value below 0.4 showed the strongest association with immune dysfunction, which also concerned NK+ cells. Senescent cells have numerous defects including a decreased capacity for proliferation, an inability to produce cytokines, short telomeres and low telomerase activity (18).

Indeed, KLRG1 plays an inhibitory role in T and NK cells and its expression typically increases with age, contributing to the inability of the immune system to respond to antigens and mount optimum responses (19). Studies targeting KLRG1 showed that not only does it serve as a marker of T-cell senescence, but it is also predictive of disease severity and cancer. Indeed, in cancer cells the dysregulation of immune checkpoint proteins is an important mechanism of tumor immune resistance and KLRG1 has been associated with both solid and hematological malignancies (19). Moreover, although KLRG-1 expression on NK cells does not necessarily imply senescence, KLRG-1+ NK cells have been associated with reduced effector functions through the activation of the AMP-protein kinase pathway, potentially rendering the host more susceptible to infections and cancer (20).

In case of CD4/CD8 ratio values below 0.4, in addition to T-cell dysfunction, we also found a decreased percentage of NK cells, which are known to display several killing abilities aiming to protect the host against infectious pathogens and malignant cells; they participate both in innate immunity and cellular responses linked to adaptive immunity (18, 19). Although we did not find a significant increase in senescent NK cells, the expression of KLRG1 on these cells has been associated with functional impairment and active HIV transcription, suggesting that its targeting may be useful to reduce the HIV reservoir (21).

Comorbid conditions were associated with increased immune dysfunction, with hypertension and osteoporosis showing the strongest immune disruption. Interestingly, CD8+ EM senescent cells were significantly associated with high blood pressure. Chronic immune activation has been postulated to be one of the culprits for hypertension in PLWH (22). Indeed, previous studies showed that innate and adaptive immunity participate to cardiovascular diseases and hypertension by the production of pro-inflammatory cytokines such as TNF-alpha, IFN-gamma and IL-17A (23). Similarly, previous studies also showed that activated and senescent CD8+ cells are associated with low bone mass density in PLWH on successful treatment (24). The increased prevalence of CD8+ EM cells among subjects with hypertension suggests that the inflammatory environment generated by HIV infection has detrimental consequences on T-cell homeostasis, with the accumulation of cells displaying an activated phenotype and higher risks for the development and the maintenance of hypertension (25).

Although we did not find any association between CD4/CD8 ratio cut-offs and cancer, the profound modifications in T-cell and the NK compartments in case of CD4/CD8 ratio below 0.4 could explain the highest risks for AIDS and non-AIDS malignancy with a ratio below 0.3 reported by others (1, 26).

The lack of increased immunosenescence among those with a history of cancer could be explained by prior but currently inactive tumors, or by the limited number of subjects with this type of comorbidity.

Limits of this study include the relative small number of subjects included and the lack of younger individuals for comparison. Indeed, we chose to include older subjects as they have higher risks for comorbid conditions and immunosenescence, but it would have been useful to investigate whether the same results could also be found among younger individuals. Moreover, immune activation markers were not measured in plasma, while the analysis of monocyte subsets could have offered other interesting insights into immune activation and immunosenescence.

In conclusion, our results show that PLWH on successful treatment with a CD4/CD8 ratio below 0.76 displayed higher levels of immunosenescence markers in T-cells, while those with a CD4/CD8 ratio below 0.4 showed the strongest degree of immune dysfunction, including a decrease in NK-cells. Such results could be useful to identify patients requiring closer monitoring for clinical progression.

## Data Availability

The original contributions presented in the study are included in the article/Supplementary material, further inquiries can be directed to the corresponding author.
