# Supplementary material for: Immunosenescence markers in T- and NK-cells according to the CD4/CD8 ratio in successfully treated people living with HIV
Source: Front Med (Lausanne). 2025 Apr 15;12:1562537. doi: 10.3389/fmed.2025.1562537 (PMC12037392; doi:10.3389/fmed.2025.1562537)
Supplement: Supplementary file 1 [file Table_1.docx]

| Supplementary Table 1: Spearman‘s Rho correlations between quantitative variables in PLWH (n=68) | | | | |
| --- | --- | --- | --- | --- |
|  |  |  |  |  |
|  |  |  |  |  |
|  | Correlation with CD4/CD8 | | Correlation with time on ART | |
|  | Spearman's Rho | p-value | Spearman's Rho | p-value |
| Time on last ART (years) | 0.01 | 0.913 | -0.12 | 0.390 |
| HIV follow-up (years) | 0.16 | 0.194 | 0.38 | 0.002 |
| Age at inclusion | -0.27 | 0.025 | 0.21 | 0.094 |
| Time on ART (years) | -0.08 | 0.529 | - | - |
| lymphocytes count (cells/microL) | 0.06 | 0.611 | 0.10 | 0.423 |
| CD3+ cells (%) | -0.30 | 0.016 | 0.21 | 0.095 |
| NK cells (%) | 0.17 | 0.168 | -0.14 | 0.277 |
| CD4+ cells (%) | 0.84 | 0.000 | -0.01 | 0.937 |
| CD8+ cells (%) | -0.90 | 0.000 | 0.17 | 0.178 |
| senescent CD8+ cells (%) | -0.50 | 0.000 | 0.05 | 0.685 |
| senescent CD4+ cells (%) | -0.32 | 0.010 | -0.06 | 0.652 |
| senescent NK cells (%) | -0.06 | 0.621 | 0.22 | 0.081 |
| NK CD56^bright^ cells (%) | -0.23 | 0.067 | 0.08 | 0.518 |
