# Supplementary material for: Immunosenescence markers in T- and NK-cells according to the CD4/CD8 ratio in successfully treated people living with HIV
Source: Front Med (Lausanne). 2025 Apr 15;12:1562537. doi: 10.3389/fmed.2025.1562537 (PMC12037392; doi:10.3389/fmed.2025.1562537)
Supplement: Supplementary file 3 [file Presentation_1.pptx]

## Slide 1
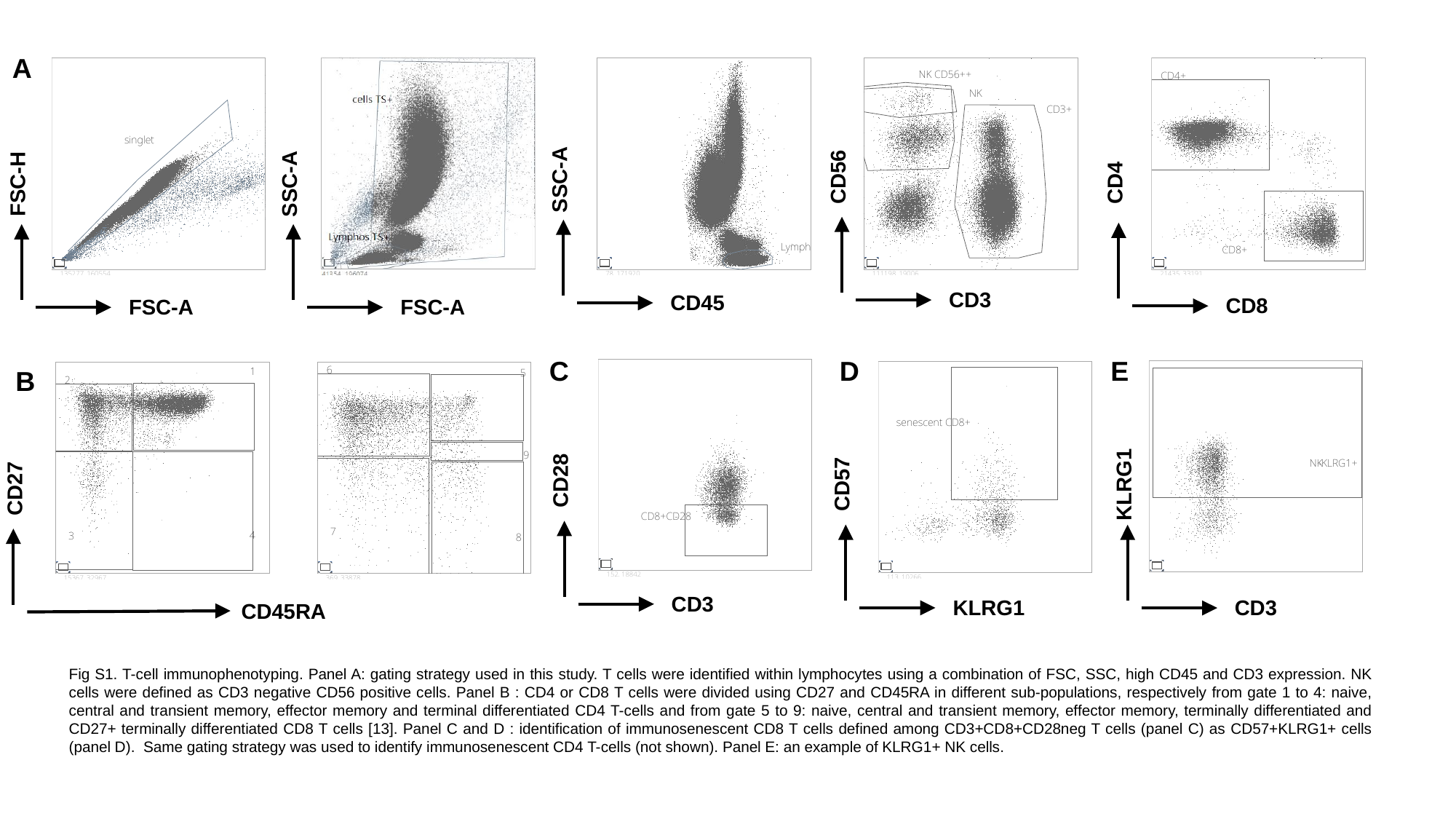

A
FSC-H
FSC-A
SSC-A
FSC-A
SSC-A
CD45
CD56
CD3
CD4
CD8
C
CD28
CD3
D
CD57
KLRG1
E
KLRG1
CD3
B
CD27
CD45RA
Fig S1. T-cell immunophenotyping. Panel A: gating strategy used in this study. T cells were identified within lymphocytes using a combination of FSC, SSC, high CD45 and CD3 expression. NK cells were defined as CD3 negative CD56 positive cells. Panel B : CD4 or CD8 T cells were divided using CD27 and CD45RA in different sub-populations, respectively from gate 1 to 4: naive, central and transient memory, effector memory and terminal differentiated CD4 T-cells and from gate 5 to 9: naive, central and transient memory, effector memory, terminally differentiated and CD27+ terminally differentiated CD8 T cells [13]. Panel C and D : identification of immunosenescent CD8 T cells defined among CD3+CD8+CD28neg T cells (panel C) as CD57+KLRG1+ cells (panel D). Same gating strategy was used to identify immunosenescent CD4 T-cells (not shown). Panel E: an example of KLRG1+ NK cells.

## Slide 2
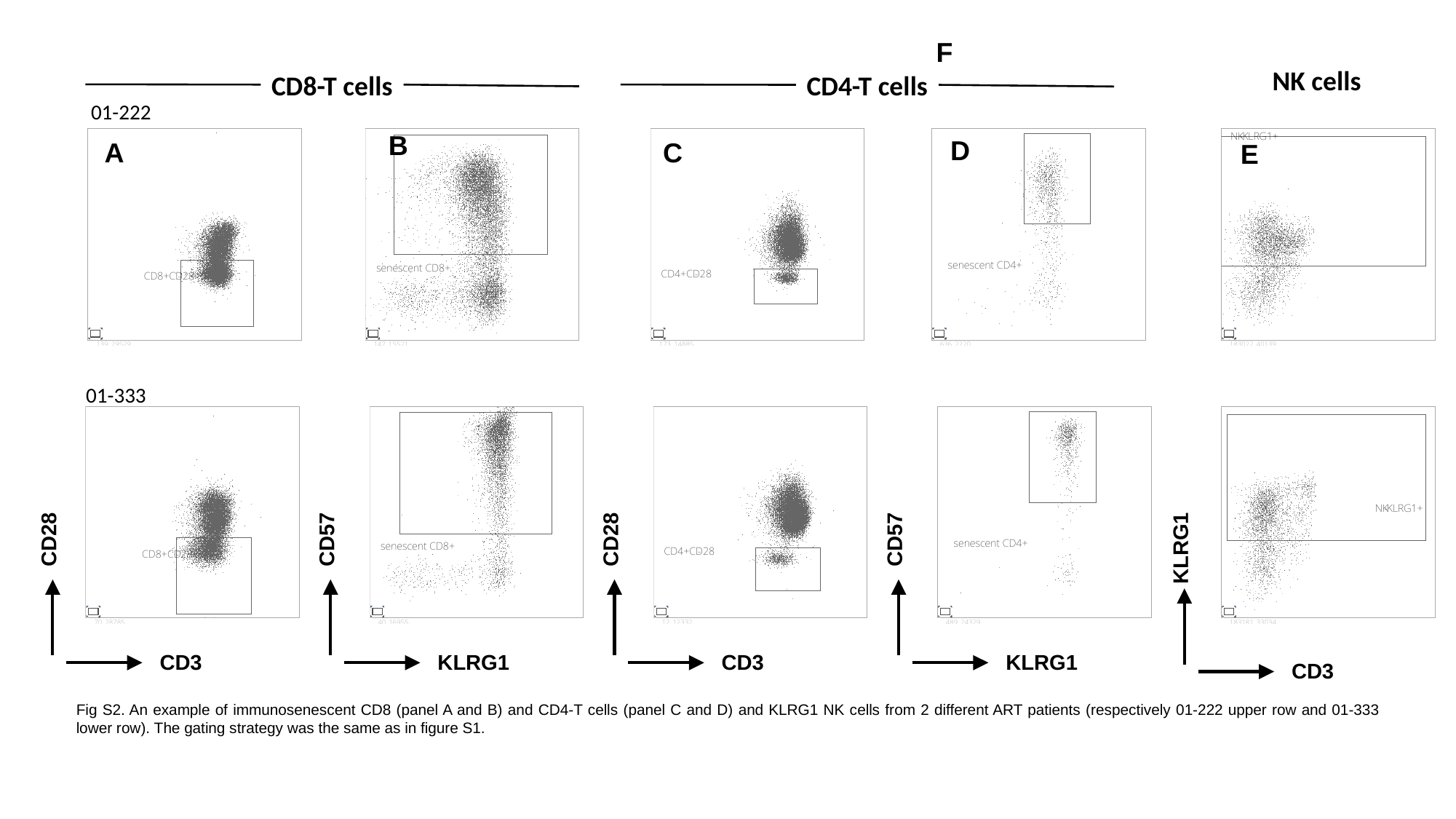

F
NK cells
CD8-T cells
CD4-T cells
01-222
B
D
A
C
E
01-333
CD28
CD3
CD57
KLRG1
CD28
CD3
CD57
KLRG1
KLRG1
CD3
Fig S2. An example of immunosenescent CD8 (panel A and B) and CD4-T cells (panel C and D) and KLRG1 NK cells from 2 different ART patients (respectively 01-222 upper row and 01-333 lower row). The gating strategy was the same as in figure S1.

## Slide 3
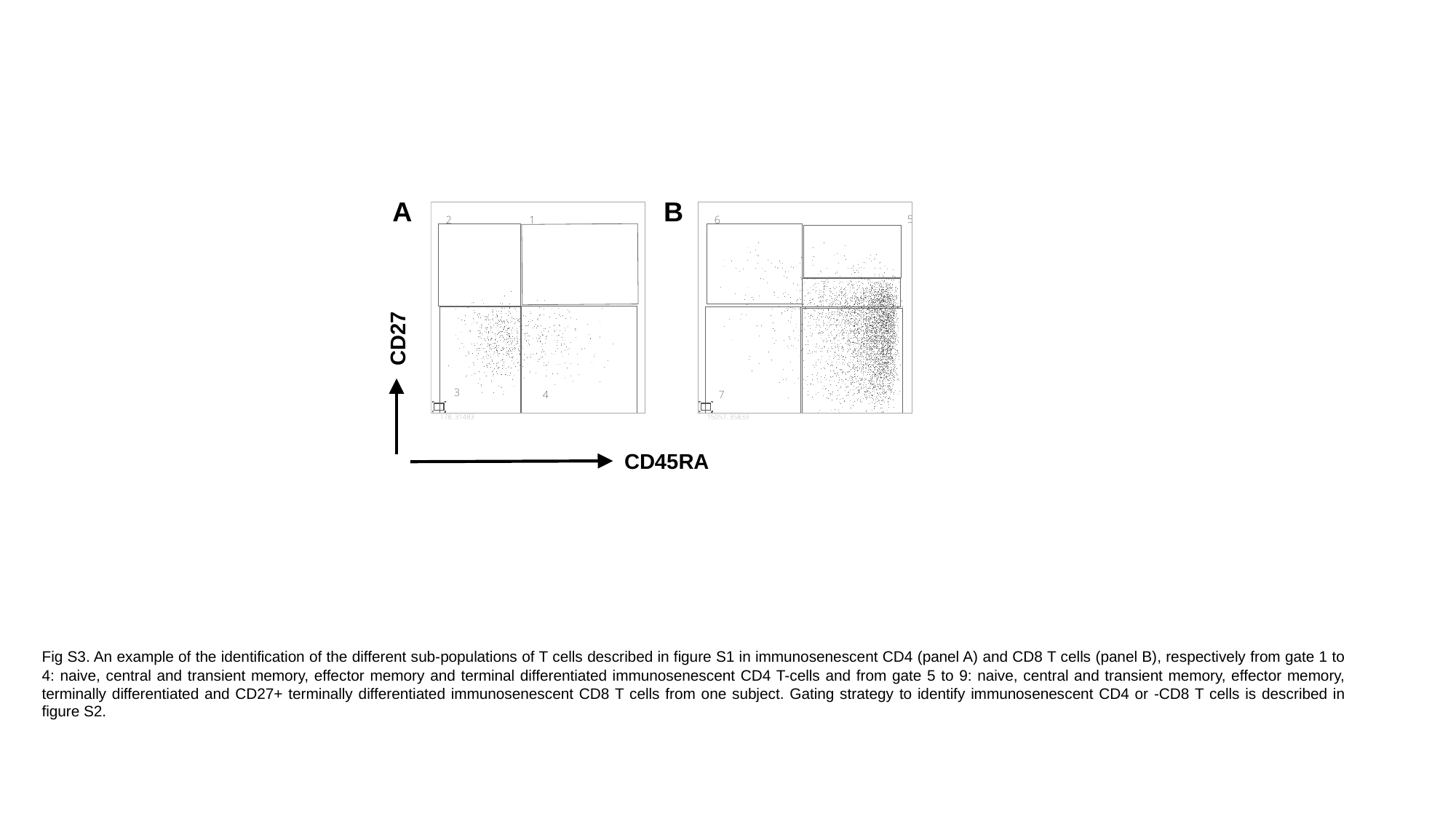

A
B
CD27
CD45RA
Fig S3. An example of the identification of the different sub-populations of T cells described in figure S1 in immunosenescent CD4 (panel A) and CD8 T cells (panel B), respectively from gate 1 to 4: naive, central and transient memory, effector memory and terminal differentiated immunosenescent CD4 T-cells and from gate 5 to 9: naive, central and transient memory, effector memory, terminally differentiated and CD27+ terminally differentiated immunosenescent CD8 T cells from one subject. Gating strategy to identify immunosenescent CD4 or -CD8 T cells is described in figure S2.
